# Effects of Low versus High Glycemic Index Sugar-Sweetened Beverages on Postprandial Vasodilatation and Inactivity-Induced Impairment of Glucose Metabolism in Healthy Men

**DOI:** 10.3390/nu8120802

**Published:** 2016-12-10

**Authors:** Judith Keller, Julia Kahlhöfer, Andreas Peter, Anja Bosy-Westphal

**Affiliations:** 1 Institute of Nutritional Medicine, University of Hohenheim, 70599 Stuttgart, Germany; judith.karschin@uni-hohenheim.de (J.K.); julia@kahlhoefers.de (J.K.); 2 Department of Internal Medicine, Division of Endocrinology, Diabetology, Angiology, Nephrology and Clinical Chemistry, University Hospital of Tübingen, 72076 Tübingen, Germany; Andreas.Peter@med.uni-tuebingen.de; 3 Institute for Diabetes Research and Metabolic Diseases of the Helmholtz Centre Munich, University of Tübingen, 72076 Tübingen, Germany; 4 German Center for Diabetes Research (DZD), 72076 Tübingen, Germany

**Keywords:** arterial stiffness, vasodilatation, GLP-1, glycemic index, sugar-sweetened beverages, low-physical activity, healthy men

## Abstract

Intake of sugar-sweetened beverages (SSB) may contribute to cardiovascular risk. The aim of this study was to investigate whether functional sugars with low compared to high glycemic index (GI) have beneficial effects on arterial stiffness during a period of low-physical activity. In a controlled cross-over dietary intervention (55% CHO, 30% fat, 15% protein), 13 healthy men (age: 23.7 ± 2.2 years, body mass index: 23.6 ± 1.9 kg/m^2^) completed 2 × 1 week of low physical activity following 1 week of normal physical activity (2363 ± 900 vs. 11,375 ± 3124 steps/day). During inactive phases participants consumed either low-GI (isomaltulose) or high-GI SSB (maltodextrin-sucrose), providing 20% of energy requirements. Postprandial vasodilatation (augmentation index, AIx), insulin sensitivity (IS) and Glucagon-like-peptide 1 (GLP-1) responses were measured during a meal test before and after SSB-intervention. Compared to maltodextrin-sucrose-SSB, postprandial vasodilatation was prolonged (AIx after 120 min: 9.9% ± 4.3% vs. 11.4% ± 3.7%, *p* < 0.05) and GLP-1 secretion was higher with isomaltulose-SSB (total area under the GLP-1 curve (tAUC_GLP_)-1: 8.0 ± 4.4 vs. 5.4 ± 3.4 pM × 3 h; *p* < 0.05). One week of low-physical activity led to impaired IS that was attenuated with low-GI SSB consumption, but did not affect arterial stiffness (*p* > 0.05). Higher postprandial GLP-1 secretion after intake of low compared to high-GI beverages may contribute to improved postprandial vasodilatation. Although one week of low-physical activity led to marked impairment in IS, it had no effect on arterial stiffness in healthy men.

## 1. Introduction

Despite recommendations to limit consumption of sugar sweetened beverages (SSB), intake has been increasing in the last decades [1]. Germany has the highest sale numbers of SSBs, compared to other European countries [2]. The corresponding high glycemic and insulinemic responses associated with SSB consumption not only promote weight gain and contribute to increased risk of type 2 diabetes [3] but may also increase blood pressure (BP) and serum lipids independent of body weight [4]. Cross-sectional studies indicate direct associations between SSB consumption and BP as well as cardiovascular risk factors, such as low density lipoprotein (LDL) cholesterol and triglycerides [5,6,7]. Weight-loss induced improvement in BP is also better with a low glycemic load diet compared to a low fat diet (43% CHO and glycemic index, GI = 50 vs. 65% CHO and GI = 82) [8] and decrease in pulse wave velocity and 24-h BP was found with a 6-month low-GI diet intervention but not with a high-GI diet [9].

Although it is suggested that greater SSB consumption may contribute to the development of hypertension, adverse lipid parameters and coronary heart disease, intervention studies investigating the effect of SSB consumption on arterial stiffness are lacking [10].

Because hyperglycemia can lead to insulin resistance and thus to endothelial dysfunction [11] a diet with a high glycemic load may be causally related to hypertension. In insulin sensitive subjects, insulin-signaling pathways lead to an activation of endothelial nitric oxide (NO) synthase via phosphatidylinositol 3-kinase and Akt and thus increase the bioavailability of the vasodilating NO in endothelial cells [12,13]. In line with this hypothesis, insulin-mediated vasodilatation is impaired with insulin resistance [11] and increased BP [14]. 

Compared to conventional sugar, SSBs sweetened with functional low-GI sugar types had beneficial effects on low-physical activity-induced impairment of glucose metabolism [15] and may therefore reduce BP. Isomaltulose (Palatinose™) and sucrose are both disaccharides consisting of glucose and fructose, providing the same number of calories but differing in their glycosidic bond. In isomaltulose the molecules are linked α-1,6 instead of α-1,2 (in sucrose) which results in slower absorbability [16] and thus a lower GI in isomaltulose than in sucrose (GI: 32 for isomaltulose vs. 64 for sucrose) [17]. 

Compared to beverages sweetened with maltodextrin-sucrose (e.g., commonly used in sport drinks [18], beverages sweetened with functional low-GI sugar isomaltulose had beneficial effects on inactivity-induced impairment of glucose metabolism [15] and thus insulin-mediated vasodilatation. By contrast, acute effects of postprandial relaxation of the vascular wall may be lower with isomaltulose compared to sucrose because of lower postprandial insulin secretion. However, the intake of low-GI isomaltulose not only resulted in lower insulin levels but also in a higher Glucagon-like-peptide 1 (GLP-1) response [19] and recent studies have shown that the incretin hormone GLP-1 has beneficial effects on endothelium-dependent vasodilatation in humans [20,21]. Despite lower insulin secretion after low-GI isomaltulose SSB intake, postprandial vasodilatation may therefore be similar or even higher compared to high-GI SSB due to a higher postprandial GLP-1 response.

The present study investigates the effect of acute and chronic (one week) intake of high- vs. low-GI SSB on arterial stiffness in young healthy subjects. In contrast to the proposed effects of SSB consumption, physical inactivity is known to impair insulin sensitivity [22] and endothelial function [23,24] and thus represents a metabolic condition vulnerable for the adverse effects of SSB consumption. Impaired insulin sensitivity has been shown to be an early response to inactivity with a reduction of insulin action already occurring within one day of prolonged sitting [25]. We therefore hypothesized that one-week consumption of low-GI compared to high-GI SSBs at a low level of physical activity leads to a better maintenance of insulin sensitivity and thus to lower impairment of low physical activity-induced endothelial function.

## 2. Materials and Methods

Sixteen healthy young men were recruited at the campus of the University of Stuttgart and Hohenheim between August 2014 and February 2015. All subjects completed a medical history and a physical examination to assess health status. Inclusion criteria were: high level of physical activity (active lifestyle with >10,000 steps/day and exercise workload ≥ 5 times per week) and male sex. Participants did not use any medication on a daily basis. Exclusion criteria for enrolment included chronic disease, smoking, food allergies or special diets (e.g., vegetarian). Three subjects were excluded from analysis due to lack of compliance with the study protocol. The study protocol was approved by the ethics committee of the Medical council of Baden-Württemberg, Germany, in August 2014 (ethic approval code: F-2014-072; trial registration in ClinicalTrials.gov: NCT02321033). All participants provided informed written consent before participation according to the Declaration of Helsinki. Based on the data of Krogh-Madsen et al., at 80% power and an alpha level of 5%, 10 participants per group were needed to detect a 12% difference in insulin sensitivity. In order to account for possible drop-out, we included 16 volunteers [22]. Precision of pulse wave velocity (PWV) and aortal BP (aBP) was determined in a group of five individuals (coefficient of variation (CV): CV_PWV_: 3.2%; CV_aBP_: 6.8%). It was calculated that a difference of 6% in PWV and aBP could be detected with a power of 80% and 5% significance level in 13 subjects.

### 2.1. Study Protocol

**Figure 1:**
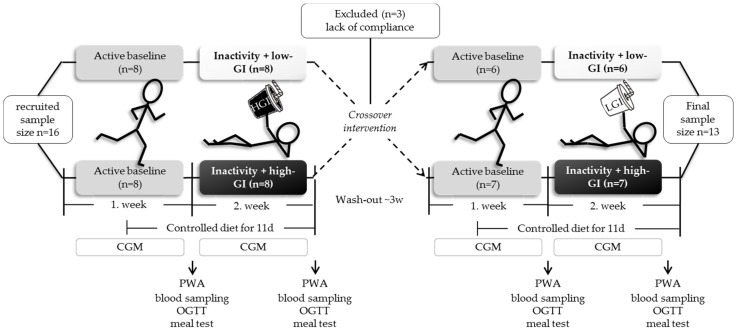
Flow diagram of the study protocol with recruitment and randomization process. During low-physical activity with GI interventions, habitual high physical activity (>10,000 steps/day) was restricted to <3000 steps/day; CGM: continuous glucose monitoring; GI: glycemic index; high-GI (HGI): beverages sweetened with a mixture of maltodextrin (75%) and sucrose (25%); low-GI (LGI): beverages sweetened with isomaltulose; meal test: 37 g isomaltulose vs. 28 g maltodextrin + 9 g sucrose; OGTT: oral glucose tolerance test; PWA: pulse wave analysis.

An outline of the study protocol is shown in the flow diagram in Figure 1. The 2 × 2 week controlled cross-over dietary intervention was carried out at the Institute of Nutritional Medicine at the University of Hohenheim. 

Over a period of two weeks, all subjects underwent one week of habitual physical activity (=active baseline) followed by one week of low-physical activity (<3000 steps/day) with either low-GI (LGI, isomaltulose) or high-GI (HGI, maltodextrin-sucrose) sugar-sweetened beverage intake, providing 20% of energy requirements. Energy requirement during the inactive period was estimated by multiplying resting energy expenditure (measured by indirect calorimetry, Quark QMR, Cosmed GmbH, Rome, Italy) by a physical activity level of 1.4, that resembles sedentary behavior. Physical activity was monitored throughout the study using the ActivPAL device (PAL Technologies Ltd., Glasgow, UK). Wash out period between low-GI and high-GI intervention was at least three weeks.

### 2.2. Diet Intervention

The controlled diet (55% CHO, 30% fat and 15% protein) started at the fourth day in the active baseline period and was maintained for 11 days (until the end of the inactive intervention period). Returned leftovers were weighted to calculate exact dietary intake. During the inactive phase, 55% CHO intake was stratified to 35% from solid food and 20% was provided as sugar-sweetened beverages with either low-GI isomaltulose (Palatinose™, provided by Beneo GmbH, Mannheim, Germany; GI = 32) or high-GI maltodextrin-sucrose (mixture of 75% maltodextrin (Nutricia GmbH, Erlangen, Germany) and 25% sucrose, adapted for sweetness; GI = 90). A detailed description of the diet is given elsewhere [15] and dietary intake, stratified by activity, low-GI and high-GI intervention is shown in Supplemental Table S1. Total amount of isomaltulose or maltodextrin-sucrose intake was 139 ± 13 g/day, respectively. Participants were instructed to consume SSBs in between meals three times per day. The beverages were provided in a single-blind randomized order.

### 2.3. Physical Activity

Physical activity was continuously monitored using the triaxial activity monitor ActivPAL^TM^ (PAL Technologies Ltd., Glasgow, UK). The device was fixed on the upper thigh with waterproof patches and was worn during the active baseline and inactive intervention periods. Step counts per day were used to define physical activity. During the active baseline periods steps should exceed >10,000 steps/day, which is recommended for healthy lifestyle in adults [26]. During low physical activity, steps were limited to <3000 steps/day to induce a vulnerable metabolic situation in healthy men. Sedentary behavior is defined as any waking behavior characterized by energy expenditure ≤ 1.5 metabolic equivalents (METs) like viewing television, video game playing, computer use, driving automobiles, and reading. By contrast, inactive behavior describes insufficient amounts of moderate- to vigorous-intensity physical activity (i.e., not meeting specified physical activity guidelines) [27]. The distinction between the two is important because a person can be sedentary throughout the entire day but at the same time physically active when engaging in exercise for 1 h in the evening. In the present study the participants were instructed to abstain from any activity >1.5 MET and spend their day sitting at desks or watching TV and they were also not allowed to do any exercise. According to the definitions the participants were therefore both, sedentary and inactive. Throughout the paper we use the term “low physical activity” for simplification.

### 2.4. Body Composition

Height was measured to the nearest 0.1 cm by stadiometer (seca 285, seca GmbH & Co. KG, Hamburg, Germany). Body weight and fat mass were measured on a calibrated impedance scale (seca mBCA 515, seca GmbH & Co. KG, Hamburg, Germany). Measurements were performed before breakfast, in underwear, without shoes and after voiding.

### 2.5. Blood Pressure, Pulse Wave Analysis and Pulse Wave Velocity (PWV)

Brachial BP and pulse wave analysis were conducted in a supine position using the Vicorder device (SMT medical, Würzburg, Germany). BP was measured after at least 5 min rest on the right upper arm in a quiet room. The average of the last two of three measurements was used for further analysis.

Heart rate (HR), central BP (cBP) and augmentation index (AIx) were analyzed according to manufactures guidelines for pulse wave analysis (Vicorder, SMT medical, 2009). After a normal BP measurement the cuff was inflated to diastolic BP and waveforms were recorded over at least 10 consecutive heartbeats, free from arrhythmia, to pick up the pulse wave. Heart rate was measured and AIx and cBP were estimated with the inbuilt and evaluated algorithm [28].

Repeatability of pulse wave velocity (PWV) measurement using the Vicorder device, was previously tested in an elderly population with high repeatability by McGreevy et al. [29]. Simultaneous measured waveforms were recorded by a volume displacement technique, using blood pressure cuffs placed around the upper thigh and neck (over the arteria carotis), on the right body site. Standard path length (L) indicated by the Vicorder instruction guidelines is the distance from the suprasternal notch to the middle of the thigh cuff. The cuffs were inflated to 60 mmHg, and high quality waveforms were recorded for 10 heartbeats with the patient lying in supine position with a 30° elevated head and shoulder. The measurement was stopped manually by the operator. The Vicorder software automatically calculates transit time (∆t) between carotid and femoral pulse waves. PWV was calculated as path length divided by the transit time of the pulse wave (PWV = L/∆t (m/s)) [30]. Two PWV readings were taken and an average value was documented.

BP, pulse wave analysis and PWV were assessed at the active baseline and the end of the inactive intervention period. To measure acute effects of sugar-sweetened beverage intake on cardiovascular dynamics, measurements were also conducted during a meal test with low- and high-GI SSB at 0, 30, 60, 90, 120 and 180 min after SSB intake.

### 2.6. Fasting and Postprandial Glucose Metabolism

Daylong interstitial glycemia and glucose variability was measured using continuous glucose monitoring system Dexcom G4 (Nintamed GmbH & Co. KG, Mainz, Germany). The device was continuously worn during both active and inactive phases. Glycemia over 24 h was determined as total area under the curve (tAUC). Glucose variability was assessed using mean amplitude of glycemic excursion (MAGE):

(1) $$\sum \frac{\lambda }{x}\;if\;\lambda >y\;$$

where *λ* is the difference from peak to nadir, *x* is the number of valid observations, and *y* is 1 SD of mean glucose in a 24-h period [31,32]. Daylong insulin secretion was assessed by 24-h c-peptide excretion at the active baseline and the end of the inactive intervention phases. 

Fasting blood samples were collected after an overnight fast (≥10 h) at the active baseline and the end of the inactive phase to assess fasting insulin sensitivity (IS) using homeostasis model assessment index (HOMA-IR): fasting glucose (mmol/L) × fasting insulin (µU/L)/22.5 [33]. Participants underwent a standard oral glucose tolerance test (OGTT, intake of 75 g glucose) to assess postprandial IS by Matsuda whole-body IS Index (Matsuda-ISI): 10,000/(√ (fasting glucose × fasting insulin) × (mean glucose × mean insulin during OGTT) [34]. 

Meal tests with low- and high-GI SSBs were performed with either 37 g isomaltulose or 28 g maltodextrin + 9 g sucrose sweetened test drink. Venous blood was sampled at 0, 30, 60, 90, 120 and 180 min after sugar consumption. Insulin, glucose and GLP-1 (only during meal test) responses were determined and calculated as incremental AUC (iAUC) or total AUC (tAUC) using the trapezoid rule [35].

### 2.7. Blood Sampling and Analytical Methods

Blood sampling was conducted by vein cannula. Serum insulin was determined by photometry (Beckman Coulter, Brea, CA, USA). Glucose was measured using hexokinase method (Beckman Coulter, Brea, CA, USA). Blood samples to analyze active GLP-1 were withdrawn directly into BD P800 plasma tubes (Becton Dickinson, Heidelberg, Germany) containing an inhibitory mix (i.e., Dipeptidyl peptidase 4 inhibitor), kept in ice and centrifuged in a refrigerated centrifuge within 10 min. Aliquots were stored at −80 °C. Circulating levels of active GLP-1 (7-36) were quantified at the University of Tübingen using the chemiluminescent high sensitivity GLP-1 Active ELISA Kit (#EZGLPHS-35K, Merck Millipore, Darmstadt, Germany). Measurements were performed in duplicate on the Glo-Max Luminometer (Promega GmbH, Mannheim, Germany) applying the five-parameter logistic (GraphPad Prism Software, La Jolla, CA, USA) according to the manufacturer’s instructions. Urinary c-peptide was analyzed with luminescence immunoassay.

### 2.8. Statistical Analysis

Data are presented as means ± SD, unless otherwise stated. Analyses were performed using SPSS version 22.0 (SPSS Inc., Chicago, IL, USA). Normal distribution was assessed by Kolmogorov-Smirnov test. The effect of activity or GI intervention or activity × GI was tested using two-way ANOVA followed by Bonferroni post hoc tests. *p*-values of significant differences between active baseline and the corresponding inactive phase were subsequently analyzed using two-tailed paired *t*-test. Baseline values for GLP-1 during meal test were considered as a covariate using repeated measures analysis of covariance (RM-ANCOVA). *p* values < 0.05 were considered statistically significant. 

## 3. Results

Baseline characteristics and changes in body weight, fat mass and lipid profile with low-physical activity and diet intervention are summarized in Table 1. At baseline, body mass index (BMI) ranged between 20.8 and 28.0 kg/m^2^, with two participants being overweight (BMI: 25.9 and 28.1 kg/m^2^). Physical activity was significantly reduced during both inactive phases, with no difference between low-GI and high-GI interventions (Δ low-GI: −80%, Δ high-GI: −79%, *p* > 0.05). During low physical activity with the high-GI SSB intervention a slight weight loss (Δ high-GI: −0.5 ± 0.7 kg) was observed whereas fat mass increased with both inactive phases without a difference between low and high-GI SSB interventions. Triglycerides (TG) and cholesterin/high density lipoprotein (HDL)-ratio increased, and HDL decreased with low physical activity with no difference between GI interventions (Table 1).

### 3.1. Effect of Low vs. High-GI SSB Intake on Postprandial GLP-1 Secretion and Parameters of Arterial Stiffness

**Figure 2:**
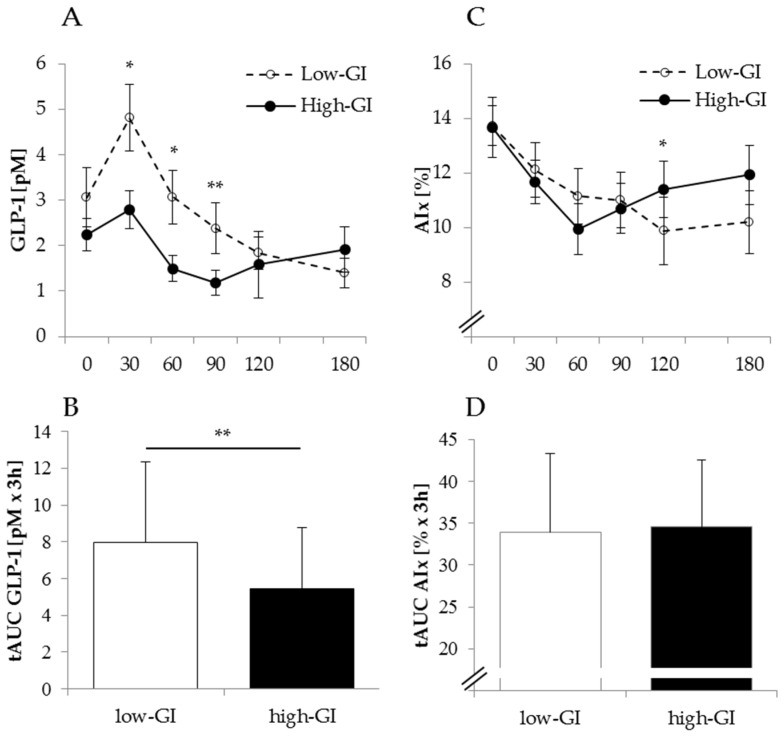
Comparison of postprandial Glucagon-like-peptide 1 (GLP-1) (**A**,**B**) levels and augmentation index (**C**,**D**) after intake of low glycemic index (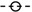) vs. high glycemic index (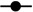) SSB at the end of one week of low physical activity. AIx, augmentation index; GLP1, glucagon-like peptide-1; SSB: sugar-sweetened beverages; tAUC, total area under the curve; upper panel: mean ± SEM, lower panel: mean ± SD; differences between tAUC for low-GI and high-GI SSB were tested using paired *t*-test, * *p* < 0.05; ** *p* < 0.01.

GLP-1 response and change in augmentation index after intake of 37 g low-GI isomaltulose vs. 37 g high-GI maltodextrin-sucrose SSB at the end of the inactive condition are shown in Figure 2. Postprandial GLP-1 levels were higher 30, 60 and 90 min after low-GI compared to high-GI SSB intake (Figure 2A). tAUC of postprandial GLP-1 response was significantly higher with low-GI compared to high-GI SSB intake (Figure 2B). Baseline fasting GLP-1 levels tended to be higher with low-GI intervention (low-GI: 3.1 ± 2.4 pM vs. high-GI: 2.2 ± 1.3 pM, *p* = 0.31), but GLP-1 response (tAUC) remained significant after controlling for these differences (RM-ANCOVA, *p* < 0.05). Postprandial vasodilatation shown as augmentation index (Figure 2C) was prolonged with low-GI compared to high-GI SSB (AIx after 120 min: 9.9% ± 4.3% vs. 11.4% ± 3.7%, *p* < 0.05), whereas tAUC of the augmentation index was not different (Figure 2D).

### 3.2. Effect of Low-GI vs. High-GI Intervention on Low Physical Activity-Induced Changes in Glucose Homeostasis

**Figure 3:**
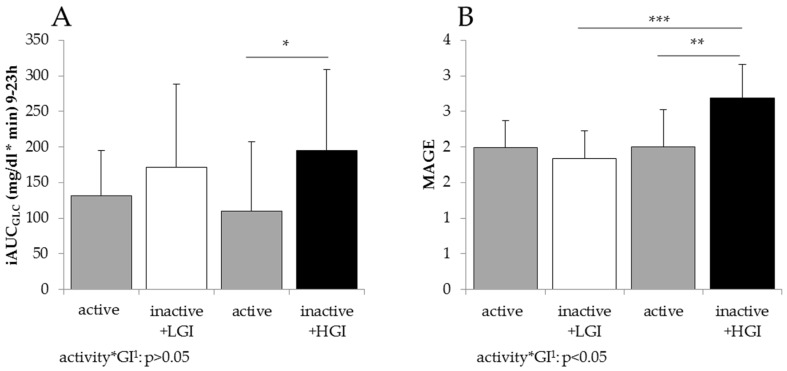
Daytime glycemia (iAUC_GLC_, **A**) and glucose variability (MAGE, **B**) during active and inactive phases with low-GI and high-GI SSB intervention. iAUC: incremental area under the curve; MAGE: mean amplitude of glycemic excursions; low-GI (LGI): beverages sweetened with isomaltulose; high-GI (HGI): beverages sweetened with a mixture of maltodextrin (75%) and sucrose (25%); mean ± SD; two-way ANOVA with Bonferroni adjustment and paired *t*-test, * *p* < 0.05; ** *p* < 0.01, *** *p* < 0.001.

Changes in insulin sensitivity and insulin secretion due to low physical activity with low and high-GI SSB intervention are shown in Table 2 and Figure 3. Fasting insulin and HOMA-IR significantly increased and Matsuda-ISI decreased due to low physical activity with both GI interventions. These impairments in fasting and postprandial insulin sensitivity were significantly lower with the low-GI compared to the high-GI SSB intervention (Table 2). The 24-h c-peptide excretion (Table 2), daytime glycemia (iAUC between 9 h and 23 h) and glucose variability (MAGE) (Figure 3), were maintained with low physical activity at the low-GI SSB intervention but increased with low physical activity at the high-GI SSB intervention (daytime glycemia: Δ low-GI: 40 ± 121; Δ high-GI: 84 ± 129 mg/dl*min; MAGE: Δ low-GI:−0.2 ± 0.5; Δ high-GI: 0.7 ± 0.6, both Δ high-GI *p* < 0.05). MAGE was significantly lower with low physical activity + low-GI compared to low physical activity + high-GI (low-GI: 1.8 ± 0.4; high-GI: 2.7 ± 0.5, *p* < 0.05, Figure 3B). While there was no GI or activity × GI effect in daytime glycemia, MAGE was affected by GI and activity × GI (*p* < 0.05 tested with two-way ANOVA, Figure 3).

### 3.3. Impact of Low-GI vs. High-GI SSB Intake on Low Physical Activity-Induced Changes in BP and Parameters of Arterial Stiffness

Changes in BP and parameters of arterial stiffness due to low physical activity stratified by GI intervention are summarized in Table 3. All parameters (systolic BP, diastolic BP, central BP, AIx and PWV) remained unchanged with low physical activity and did not differ between both interventions.

## 4. Discussion

Regarding the acute effect of SSB intake on postprandial relaxation of the vascular wall, vasodilatation was similar with low-GI isomaltulose compared to high-GI maltodextrin-sucrose intake. This equal effect occurred despite a lower insulin secretion with isomaltulose intake and might be explained by higher postprandial GLP-1 levels that may also contribute to postprandial vasodilatation. In the long term, one week of low physical activity led to impaired basal and postprandial insulin sensitivity that were attenuated with low GI compared to high GI SSB consumption, but did not affect arterial stiffness.

### 4.1. Acute Effect of Low- vs. High-GI SSB Intake on Insulin, GLP-1 and Associated Endothelial Function

The present study has shown that compared to high-GI SSB intake, low-GI SSB intake led to higher GLP-1 secretion with slightly prolonged but no difference in total postprandial vasodilation (Figure 2). Previously, we have shown that postprandial insulin response (iAUC as well as tAUC) was significantly lower with low-GI compared to high-GI SSB consumption [15]. A 50% lower insulin secretion with isomaltulose compared to sucrose intake has been shown in healthy men [36], and was explained by slower digestion of isomaltulose compared to sucrose [16]. Although GLP-1 response has been shown to be lower after pure fructose intake when compared with an equicaloric glucose load [37] SSB intake with isomaltulose that contains 50% fructose led to significantly higher GLP-1 release compared to maltodextrin-sucrose intake with only 12.5% fructose (Figure 2). This indicates that a higher GLP-1 release with isomaltulose compared to maltodextrin-sucrose intake results from a slower degradation of the α-1,6 glycosidic bond of isomaltulose that leads to retarded resorption and a higher proportion of glucose reaching the distal part of the ileum where GLP-1 secreting L-cells are found [16,38]. Our findings are in line with the result of a recent study which has shown higher GLP-1 secretion with isomaltulose compared to sucrose intake in healthy men [19]. Postprandial vasodilatation is mediated by insulin signaling due to insulin-induced release of nitric oxide [39]. However, lower insulin response with isomaltulose intake resulted in similar vasodilatation compared to high insulin responses with maltodextrin-sucrose intake (Figure 2). Since it has been demonstrated that GLP-1 per se has direct beneficial effects on endothelium-dependent vasodilatation in humans [20] higher GLP-1 secretion with low-GI isomaltulose intake may compensate lower insulin responses. 

In type 2 diabetes, postprandial GLP-1 response after a mixed meal is strongly reduced, compared to healthy subjects [40] and may therefore contribute to a higher prevalence of hypertension in these patients. Although it is not clear if an inhibition of L-cell secretion or an increase in GLP-1 degradation contribute to the lower incretin effect in type 2 diabetes, the intake of low-GI isomaltulose has been shown to increase GLP-1 secretion compared to sucrose in type 2 diabetes [41] and may therefore improve postprandial vasodilatation. 

Impaired endothelium-derived vasodilatation is a general finding in hypertension and it is suggested that defects in the NO system can induce hypertension [42]. Since vasodilatation via GLP-1 receptors in human endothelial cells also appears to be induced by increased NO production (for review see [43]), the present data suggest that consumption of SSBs with isomaltulose compared to conventional high-GI SSBs could improve postprandial vasodilatation in hypertension. 

### 4.2. Effect of GI Intervention on Low Physical Activity-Induced Changes in Glucose Metabolism and Arterial Stiffness

Low physical activity is an important risk factor for all-cause mortality [44]. Prolonged sitting of 3–6 h leads to impaired endothelial function, i.e., a lower shear rate and flow mediated vasodilatation in the superficial femoral artery [45,46] and five days of bed rest in healthy subjects results in decreased endothelial function, arterial stiffening, increased diastolic blood pressure and development of insulin resistance and dyslipidemia [24,47]. However, in the present study BP and arterial stiffness were not affected by one week of low physical activity despite impaired insulin sensitivity and lipid profile (Table 1 and Table 2).

Endothelial dysfunction also belongs to the metabolic abnormalities accompanying insulin resistance and type 2 diabetes (for review see [11]). Underlying mechanisms include the activation of protein kinase C, increased expression of adhesion molecules, production of endothelin, increased proliferation of endothelial cells and decreased production of nitric oxide (NO), e.g., induced by hyperglycemia (for review see [48]). This shift between vasodilator and vasoconstrictor actions of insulin is important for the pathophysiology of vascular dysfunction in insulin resistance [13]. Although the present data did not show impaired arterial stiffness with decreased IS in healthy men, an increase in arterial stiffness already occurs before the onset of type 2 diabetes in subjects with increased plasma glucose levels [49,50] and is also increased in obese and insulin resistant adolescents and young adults [51]. Although daylong glycemia did not show activity × GI effect, the fluctuation in glucose levels assessed by MAGE was significantly increased by high-GI SSB but not by low-GI SSB consumption during low physical activity (Figure 3). Hyperglycemia-induced vascular damage is triggered by overproduction of superoxide by the mitochondrial electron-transport chain [52]. In addition, glycemic variability has been proposed to have more deleterious effects than sustained hyperglycemia because increments and decreases in glucose levels both increase oxidative stress in patients with type 2 diabetes [53,54]. However, higher glycemic variability during high-GI SSB consumption had no effect on arterial stiffness (Table 3). This may be due to a persistent protective effect of physical fitness in our habitually very active young men (i.e., >10,000 steps per day) because training reinforces the endogenic antioxidative capacity and results in a reduction in oxidative stress and improved endothelial function [55] whereas detraining has been shown to only gradually increase oxidative stress and systolic blood pressure over time [56]. 

Intervention studies investigating the beneficial effect of low-GI diets on insulin sensitivity are limited to obese/overweight subjects with insulin resistance [57]. Our data provide evidence that intake of low-GI compared to high-GI SSB led to an attenuation of the impairment in glucose metabolism with low physical activity (Table 2). The better maintenance of glucose metabolism with low-GI isomaltulose could therefore contribute to preserve endothelial function in long-term. In line with this hypothesis, the results of a 6-month intervention indicate that a low-GI diet may be beneficial in reducing risk of coronary heart disease, including PWV and 24-h BP with concurrent lower insulin resistance compared to high-GI diet [9]. This result is supported by a 24-h intervention with a low-GI diet compared to a high-GI diet in normotensive women that has shown a lower 24-hour BP with low-GI diet [58]. In addition, 10% weight-loss with low vs. high glycemic load diets in young overweight and obese adults resulted in a higher reduction of BP [8].

### 4.3. Study Limitations

Baseline fasting GLP-1 levels tended to be higher with low-GI intervention compared to high-GI intervention (Figure 2). This may be an adaptation to the daily intake of isomaltulose-sweetened SSB during the inactive intervention period prior to the measurement. The ratio of glucose:fructose differed between both SSB interventions. Low-GI SSB with isomaltulose had a ratio of 50:50, whereas high-GI SSB with maltodextrin + sucrose had a glucose:fructose ratio of 87.5:12.5. Since our study only covers a short intervention period of one week in healthy people, the results are not transferable to chronic effects and groups of patients. The long-term effects of low-GI sugars on endothelial function and glucose metabolism therefore need to be investigated in subjects with impaired glucose tolerance and/or hypertension.

## 5. Conclusions

One week of low physical activity led to impaired insulin sensitivity, while arterial stiffness was not affected. Positive effects of postprandial GLP-1 secretion with isomaltulose compared to maltodextrin-sucrose intake may contribute to postprandial vasodilatation. 

## Figures and Tables

**Table 1:** Basal characteristics and low physical activity-induced changes of body weight, fat mass and cardiovascular risk factors stratified by low-GI vs. high-GI SSB intervention, *n* = 13.

	Activity 1	Low Physical Activity + Low-GI	Activity 2	Low Physical Activity + High-GI	∆ Low Physical Activity + Low-GI—Activity 1 ^1^	∆ Low Physical Activity + High-GI—Activity 2 ^1^	Difference Between Changes Upon Low Physical Activity ^2^
Age	24 ± 7						
BMI (kg/m^2^)	23.4 ± 2.3						
Weight	78.8 ± 9.8	78.7 ± 9.8	78.7 ± 9.2	78.2 ± 9.3	−0.1 ± 0.6	−0.5 ± 0.7 *	n.s.
FM (%)	13.8 ± 6.4	14.3 ± 6.2	13.4 ± 5.7	14.1 ± 5.6	0.5 ± 0.8 *	0.7 ± 0.7 **	n.s.
Steps per day	11,051 ± 3064	2263 ± 861	11,699 ± 3748	2463 ± 1008	−8788 ± 3131 ***	−9236 ± 3940 ***	n.s.
**Lipid profile**							
TG (mg/dL)	63.2 ± 22.1	94.7 ± 28.1	65.3 ± 17.2	115.3 ± 52.6	31.5 ± 36.4 *	50.0 ± 53.1 **	n.s.
Cholesterin (mg/dL)	152 ± 24	157 ± 23	156 ± 24	156 ± 28	5.5 ± 16.9	−0.2 ± 13.1	n.s.
HDL (mg/dL)	45.8 ± 6.2	41.5 ± 7.0	47.4 ± 7.0	42.2 ± 7.4	−4.2 ± 5.7 *	−5.2 ± 4.3 **	n.s.
LDL (mg/dL)	93.9 ± 17.4	100.6 ± 15.5	97.6 ± 16.7	99.4 ± 21.8	6.7 ± 12.4	1.8 ± 10.4	n.s.
Cholesterin/HDL	3.4 ± 0.6	3.8 ± 0.7	3.3 ± 0.6	3.8 ± 0.8	0.5 ± 0.4 **	0.4 ± 0.5 **	n.s.

GI: glycemic index; low-GI: isomaltulose; high-GI: mixture of 75% maltodextrin and 25% sucrose; SSB: sugar-sweetened beverage; BMI: body mass index; FM: fat mass; n.s.: not significant; TG: triglycerides; HDL: high density lipoptrotein; LDL: low density lipoprotein; ^1^ difference between active and inactive period; ^2^ difference between changes upon low physical activity with low-GI and high-GI SSB (activity × GI); * *p* < 0.05, ** *p* < 0.01, *** *p* < 0.001, two-way ANOVA with Bonferroni adjustment; paired *t*-test.

**Table 2:** Comparison of low physical activity-induced changes in fasting insulin, insulin sensitivity (IS), C-peptide excretion, daylong glycemia and glucose variability between low-GI and high-GI SSB intervention. *n* = 13.

	Activity 1	Low Physical Activity + Low-GI	Activity 2	Low Physical Activity + High-GI	∆ Low Physical Activity + Low-GI—Activity 1 ^1^	∆ Low Physical Activity + High-GI—Activity 2 ^1^	Difference between Changes upon Low Physical Activity ^2^
Fasting insulin (mU/mL)	4.1 ± 2.2	6.0 ± 2.6	3.8 ± 1.8	7.7 ± 4.9	1.9 ± 2.4 *	3.9 ± 3.6 **	<0.05
HOMA-IR	0.8 ± 0.4	1.2 ± 0.7	0.7 ± 0.5	1.6 ± 1.2	0.4 ±0.5 **	0.8 ± 0.9 *	<0.05
Matsuda-ISI	14.5 ± 5.9	9.4 ± 3.4	17.4 ± 8.0	7.8 ± 3.6	−5.1 ± 5.5 *	−9.6 ± 5.1 **	<0.01
24-h C-peptide excretion (µg/day)	40.6 ± 20.7	45.0 ± 15.1	41.7 ± 18.3	64.8 ± 24.3	4.4 ± 15.1	23.1 ± 22.5 **	<0.05

GI: glycemic index; low-GI: isomaltulose; high-GI: mixture of 75% maltodextrin and 25% sucrose; SSB: sugar-sweetened beverage; HOMA-IR: homeostasis model assessment of insulin resistance; Matsuda-ISI: Matsuda insulin sensitivity index; ^1^ difference between active and inactive period; ^2^ difference between changes upon low physical activity with low-GI and high-GI SSB (activity × GI); * *p* < 0.05, ** *p* < 0.01, *** *p* < 0.001, two-way ANOVA with Bonferroni adjustment; paired *t*-test.

**Table 3:** Comparison of low physical activity-induced changes in blood pressure and parameters of arterial stiffness between low-GI and high-GI SSB intervention. *n* = 13.

	Activity 1	Low Physical Activity + Low-GI	Activity 2	Low Physical Activity + High-GI	∆ Low Physical Activity + Low-GI—Activity 1 ^1^	∆ Low Physical Activity + High-GI—Activity 2 ^1^	Difference between Changes upon Low Physical Activity ^2^
sBP (mmHg)	126 ± 8	124 ± 6	124 ± 8	125 ± 6	−1.8 ± 6.9	0.8 ± 5.5	n.s.
dBP (mmHg)	66 ± 5	65 ± 4	65 ± 5	64 ± 6	−1.0 ± 3.8	−0.4 ± 6.3	n.s.
cBP (mmHg)	119 ± 8	117 ± 6	116 ± 7	117 ± 6	−2.3 ± 5.9	0.6 ± 4.8	n.s.
AIx (%)	14 ± 3	14 ± 3	13 ± 4	14 ± 4	0.0 ± 2.9	0.1 ± 3.0	n.s.
PWV (m/s)	6.5 ± 0.6	6.5 ± 0.4	6.4 ± 0.6	6.6 ± 0.4	0.0 ± 0.3	0.2 ± 0.4	n.s.

GI: glycemic index; low-GI: isomaltulose; high-GI: mixture of 75% maltodextrin and 25% sucrose; n.s.: not significant; SSB: sugar-sweetened beverage; sBP: systolic blood pressure (brachial); diastolic blood pressure (brachial); cBP: central blood pressure; Aix: augmentation index; PWV: pulse wave velocity (carotid-femoral); ^1^ difference between active and inactive period; ^2^ difference between changes upon low physical activity with low-GI and high-GI SSB (activity × GI); two-way ANOVA with Bonferroni adjustment; paired *t*-test.

## Supplementary Materials

The following are available online at http://www.mdpi.com/2072-6643/8/12/802/s1, Table S1: Composition of the diet stratified by activity, low-GI and high-GI intervention.

## References

[1] World Health Organization (2014). Reducing Consumption of Sugar-Sweetened Beverages to Reduce the Risk of Unhealthy Weight Gain in Adults. http://www.who.int/elena/bbc/ssbs_adult_weight/en/.

[2] Popkin B.M., Hawkes C. (2015). Sweetening of the global diet, particularly beverages: Patterns, trends, and policy responses. Lancet Diabetes Endocrinol..

[3] Malik V.S., Hu F.B. (2012). Sweeteners and risk of obesity and type 2 diabetes: The role of sugar-sweetened beverages. Curr. Diabetes Rep..

[4] Te Morenga L.A., Howatson A.J., Jones R.M., Mann J. (2014). Dietary sugars and cardiometabolic risk: Systematic review and meta-analyses of randomized controlled trials of the effects on blood pressure and lipids. Am. J. Clin. Nutr..

[5] Brown I.J., Stamler J., van Horn L., Robertson C.E., Chan Q., Dyer A.R., Huang C.C., Rodriguez B.L., Zhao L., Daviglus M.L. (2011). Sugar-sweetened beverage, sugar intake of individuals, and their blood pressure: International study of macro/micronutrients and blood pressure. Hypertension.

[6] Welsh J.A., Sharma A., Cunningham S.A., Vos M.B. (2011). Consumption of added sugars and indicators of cardiovascular disease risk among US adolescents. Circulation.

[7] Duffey K.J., Huybrechts I., Mouratidou T., Libuda L., Kersting M., De Vriendt T., Gottrand F., Widhalm K., Dallongeville J., Hallström L. (2012). Beverage consumption among European adolescents in the HELENA study. Eur. J. Clin. Nutr..

[8] Pereira M.A., Swain J., Goldfine A.B., Rifai N., Ludwig D.S. (2004). Effects of a low-glycemic load diet on resting energy expenditure and heart disease risk factors during weight loss. JAMA.

[9] Philippou E., Bovill-Taylor C., Rajkumar C., Vampa M.L., Ntatsaki E., Brynes A.E., Hickson M., Frost G.S. (2009). Preliminary report: The effect of a 6-month dietary glycemic index manipulation in addition to healthy eating advice and weight loss on arterial compliance and 24-hour ambulatory blood pressure in men: A pilot study. Metabolism.

[10] Malik V., Popkin B., Bray G., Després J.-P., Hu F. (2010). Sugar Sweetened Beverages, Obesity, Type 2 Diabetes and Cardiovascular Disease risk. Circulation.

[11] Rask-Madsen C., King G.L. (2007). Mechanisms of Disease: Endothelial dysfunction in insulin resistance and diabetes. Nat. Clin. Pract. Endocrinol. Metab..

[12] Zeng G., Nystrom F.H., Ravichandran L.V., Cong L.N., Kirby M., Mostowski H., Quon M.J. (2000). Roles for insulin receptor, PI3-kinase, and Akt in insulin-signaling pathways related to production of nitric oxide in human vascular endothelial cells. Circulation.

[13] Kim J.A., Montagnani M., Kwang K.K., Quon M.J. (2006). Reciprocal relationships between insulin resistance and endothelial dysfunction: Molecular and pathophysiological mechanisms. Circulation.

[14] Feldman R.D., Bierbrier G.S. (1993). Insulin-mediated vasodilation: Impairment with increased blood pressure and body mass. Lancet.

[15] Kahlhöfer J., Karschin J., Silberhorn-Bühler H., Breusing N., Bosy-Westphal A. (2016). Effect of low-glycemic-sugar-sweetened beverages on glucose metabolism and macronutrient oxidation in healthy men. Int. J. Obes..

[16] Lina B.A.R., Jonker D., Kozianowski G. (2002). Isomaltulose (Palatinose): A review of biological and toxicological studies. Food Chem. Toxicol..

[17] Atkinson F., Foster-Powell K., Brand-Miller J.C. (2008). Glycemic Load Values: 2008. Diabetes Care.

[18] Van Nieuwenhoven M.A., Brouns F., Kovacs E.M.R. (2005). The effect of two sports drinks and water on GI complaints and performance during an 18-km run. Int. J. Sports Med..

[19] Maeda A., Miyagawa J.I., Miuchi M., Nagai E., Konishi K., Matsuo T., Tokuda M., Kusunoki Y., Ochi H., Murai K. (2013). Effects of the naturally-occurring disaccharides, palatinose and sucrose, on incretin secretion in healthy non-obese subjects. J. Diabetes Investig..

[20] Basu A., Charkoudian N., Schrage W., Rizza R A., Basu R., Joyner M.J. (2007). Beneficial effects of GLP-1 on endothelial function in humans: Dampening by glyburide but not by glimepiride. Am. J. Physiol. Endocrinol. Metab..

[21] Nyström T., Gutniak M.K., Zhang Q., Zhang F., Holst J.J., Ahrén B., Sjöholm Å. (2004). Effects of glucagon-like peptide-1 on endothelial function in type 2 diabetes patients with stable coronary artery disease. Am. J. Physiol. Endocrinol. Metab..

[22] Krogh-Madsen R., Thyfault J.P., Broholm C., Mortensen O.H., Olsen R.H., Mounier R., Plomgaard P., van Hall G., Booth F.W., Pedersen B.K. (2010). A 2-week reduction of ambulatory activity attenuates peripheral insulin sensitivity. J. Appl. Physiol..

[23] Gomez-Marcos M.A., Recio-Rodríguez J.I., Patino-Alonso M.C., Agudo-Conde C., Lasaosa-Medina L., Rodriguez-Sanchez E., Maderuelo-Fernandez J.A., García-Ortiz L., EVIDENT Group (2014). Relationship between objectively measured physical activity and vascular structure and function in adults. Atherosclerosis.

[24] Nosova E.V., Yen P., Chong K.C., Alley H.F., Stock E.O., Quinn A., Hellmann J., Conte M.S., Owens C.D., Spite M. (2014). Short-term physical inactivity impairs vascular function. J. Surg. Res..

[25] Stephens B.R., Granados K., Zderic T.W., Hamilton M.T., Braun B. (2011). Effects of 1 day of inactivity on insulin action in healthy men and women: Interaction with energy intake. Metabolism.

[26] Tudor-Locke C., Craig C.L., Brown W.J., Clemes S.A., De Cocker K., Giles-Corti B., Hatano Y., Inoue S., Matsudo S.M., Mutrie N. (2011). How many steps/day are enough? For adults. Int. J. Behav. Nutr. Phys. Act..

[27] Mark T. (2012). Standardized use of the terms “sedentary” and “sedentary behaviours”. Appl. Physiol. Nutr. Metab..

[28] Hickson S.S., Butlin M., Broad J., Avolio A.P., Wilkinson I.B., McEniery C.M. (2009). Validity and repeatability of the Vicorder apparatus: A comparison with the SphygmoCor device. Hypertens. Res..

[29] McGreevy C., Barry M., Bennett K., Williams D. (2013). Repeatability of the measurement of aortic pulse wave velocity (aPWV) in the clinical assessment of arterial stiffness in community-dwelling older patients using the Vicorder^®^ device. Scand. J. Clin. Lab. Investig..

[30] McGreevy C., Barry M., Davenport C., Byrne B., Donaghy C., Collier G., Tormey W., Smith D., Bennett K., Williams D. (2014). The effect of vitamin D supplementation on arterial stiffness in an elderly community-based population. J. Am. Soc. Hypertens..

[31] Service F.J., Molnar G.D., Rosevear J.W., Ackerman E., Gatewood L.C., Taylor W.F. (1970). Mean amplitude of glycemic excursions, a measure of diabetic instability. Diabetes.

[32] Standl E., Schnell O., Ceriello A. (2011). Postprandial Hyperglycemia and Glycemic Variability. Diabetes Care.

[33] Matthews D.R., Hosker J.P., Rudenski A.S., Naylor B.A., Treacher D.F., Turner R.C. (1985). Homeostasis model assessment: Insulin resistance and beta-cell function from fasting plasma glucose and insulin concentrations in man. Diabetologia.

[34] Matsuda M., DeFronzo R.A. (1999). Insulin sensitivity indices obtained from oral glucose tolerance testing: Comparison with the euglycemic insulin clamp. Diabetes Care.

[35] Matthews J.N., Altman D.G., Campbell M.J., Royston P. (1990). Analysis of serial measurements in medical research. BMJ.

[36] Macdonald I., Daniel J.W. (1983). The bio-availability of isomaltulose in man and rat. Nutr. Rep. Int..

[37] Kong M.F., Chapman I., Goble E., Wishart J., Wittert G., Morris H., Horowitz M. (1999). Effects of oral fructose and glucose on plasma GLP-1 and appetite in normal subjects. Peptides.

[38] Baggio L.L., Drucker D.J. (2007). Biology of Incretins: GLP-1 and GIP. Gastroenterology.

[39] Scherrer U., Randin D., Vollenweider P., Vollenweider L., Nicod P. (1994). Nitric oxide release accounts for insulin’s vascular effects in humans. J. Clin. Investig..

[40] Vilsbøll T., Krarup T., Deacon C.F., Madsbad S., Holst J.J. (2001). Reduced postprandial concentrations of intact biologically active glucagon-like peptide 1 in type 2 diabetic patients. Diabetes.

[41] Ang M., Linn T. (2014). Comparison of the effects of slowly and rapidly absorbed carbohydrates on postprandial glucose metabolism in type 2 diabetes mellitus patients: A randomized trial 1–3. Am. J. Clin. Nutr..

[42] Lind L. (2000). Endothelium-dependent Vasodilation in Hypertension: A Review. Blood Press..

[43] Ussher J.R., Drucker D.J. (2012). Cardiovascular biology of the incretin system. Endocr. Rev..

[44] Blair S.N., Kohl H.W., Paffenbarger R.S., Clark D.G., Cooper K.H., Gibbons L.W. (1989). Physical fitness and all-cause mortality: A prospective study of healthy men and women. JAMA.

[45] Restaino R.M., Holwerda S.W., Credeur D.P., Fadel P.J., Padilla J. (2015). Impact of prolonged sitting on lower and upper limb micro- and macrovascular dilator function. Exp. Physiol..

[46] Thosar S.S., Bielko S.L., Mather K.J., Johnston J.D., Wallace J.P. (2015). Effect of prolonged sitting and breaks in sitting time on endothelial function. Med. Sci. Sports Exerc..

[47] Hamburg N.M., McMackin C.J., Huang A.L., Shenouda S.M., Widlansky M.E., Schulz E., Gokce N., Ruderman N.B., Keaney J.F., Vita J.A. (2007). Physical inactivity rapidly induces insulin resistance and microvascular dysfunction in healthy volunteers. Arterioscler. Thromb. Vasc. Biol..

[48] Lefebvre P.J., Scheen A.J. (1998). The postprandial state and risk of cardiovascular disease. Diabet. Med..

[49] Henry R.M.A., Kostense P.J., Spijkerman A.M.W., Dekker J.M., Nijpels G., Heine R.J., Kamp O., Westerhof N., Bouter L.M., Stehouwer C.D. (2003). Arterial stiffness increases with deteriorating glucose tolerance status: The Hoorn study. Circulation.

[50] Ohnishi H., Saitoh S., Takagi S., Ohata J., Isobe T., Kikuchi Y., Takeuchi H., Shimamoto K. (2003). Pulse Wave Velocity as an Indicator of Atherosclerosis in Impaired Fasting Glucose. Diabetes Care.

[51] Urbina E.M., Gao Z., Khoury P.R., Martin L.J., Dolan L.M. (2012). Insulin resistance and arterial stiffness in healthy adolescents and young adults. Diabetologia.

[52] Brownlee M. (2001). Biochemistry and molecular cell biology of diabetic complications. Nature.

[53] Ceriello A., Cavarape A., Martinelli L., Da Ros R., Marra G., Quagliaro L., Piconi L., Assaloni R., Motz E. (2004). The post-prandial state in Type 2 diabetes and endothelial dysfunction: Effects of insulin aspart. Diabet. Med..

[54] Monnier L., Mas E., Ginet C., Michel F., Villon L., Cristol J.-P., Colette C. (2006). Activation of Oxidative Stress by Acute Glucose Fluctuations Compared With Sustained Chronic Hyperglycemia in Patients With Type 2 Diabetes. JAMA.

[55] Brinkmann C., Schwinger R.H.G., Brixius K. (2011). Physical activity and endothelial dysfunction in type 2 diabetic patients: The role of nitric oxide and oxidative stress. Wien Med. Wochenschr..

[56] Kilic-Erkek O., Kilic-Toprak E., Caliskan S., Ekbic Y., Akbudak I.H., Kucukatay V., Bor-Kucukatay M. (2016). Detraining reverses exercise-induced improvement in blood pressure associated with decrements of oxidative stress in various tissues in spontaneously hypertensive rats. Mol. Cell. Biochem..

[57] Colagiuri S. (2015). Health potential of a low glycaemic index diet. BMJ.

[58] Hosseininasab M., Norouzy A., Nematy M., Bonakdaran S. (2015). Low-Glycemic-Index Foods Can Decrease Systolic and Diastolic Blood Pressure in the Short Term. Int. J. Hypertens..

