# Supplementary material for: Effects of Low versus High Glycemic Index Sugar-Sweetened Beverages on Postprandial Vasodilatation and Inactivity-Induced Impairment of Glucose Metabolism in Healthy Men
Source: Nutrients. 2016 Dec 10;8(12):802. doi: 10.3390/nu8120802 (PMC5188457; doi:10.3390/nu8120802)
Supplement: Supplementary file 1 [file nutrients-08-00802-s001.pdf]

Supplementary Materials: Effects of Low vs. High Glycemic Index Sugar-Sweetened Beverages on Postprandial Vasodilatation and Inactivity-Induced Impairment of Glucose Metabolism in Healthy Men

Judith Keller, Julia Kahlhöfer, Andreas Peter and Anja Bosy-Westphal

Table S1. Composition of the diet stratified by activity, low-GI and high-GI intervention.

	Activity 1 [1]		Low Physical Activity + Low-GI [2]		Activity 2 [1]		Low Physical Activity + High-GI [2]	
	**Mean**	**SD**	**Mean**	**SD**	**Mean**	**SD**	**Mean**	**SD**
Energy intake (kJ/day)	13,769 *,†	1850	12,819 *,†	1261	13,729 *	1327	12,830 †	1084
Carbohydrates (% energy)	51 *	2	55 †	3	50 *	3	56 †	2
Fat (% energy)	31 *,†	3	31 *,†	1	33 *	2	30 †	2
Protein (% energy)	17 *	1	14 †	1	17 *	1	14 †	0
Fiber (g/day)	34 *	7	32 *	4	33 *	7	32 *	3

[1] dietary intake averaged over 4 days where participants followed their habitual physical activity; [2] dietary intake averaged over the total inactive intervention period (7 days); RM-ANOVA: means not sharing a common superscript symbol (*,†) are different at $p < 0.05$; GI: glycemic index; low-GI: isomaltulose; high-GI: mixture of 75% maltodextrin and 25% sucrose.
